# Life cycle environmental impact of novel radiative thermal management textiles in China

**DOI:** 10.1007/s11356-026-37632-z

**Published:** 2026-03-16

**Authors:** Youshan Liang, Xiaocheng Hu, Guanhua Zhang, Yu Gong, Binlin Dou, Xiaoyu Yan

**Affiliations:** 1 School of Energy and Power Engineering, University of Shanghai for Science and Technology, Shanghai, 200093 China; 2 Environment and Sustainability Institute & Engineering Department, Faculty of Environment, Science and Economy, University of Exeter, Exeter EX4 4QF, UK; 3 University of Southampton Business School, University of Southampton, Southampton, SO16 7QB UK

**Keywords:** Life cycle assessment, Environmental impact, Sustainable development, Functional textiles, Composite fibers

## Abstract

New types of textiles are being developed to meet requirements such as adjusting body surface temperature according to changes in the surrounding environment. However, these new textiles can have a significantly different impact on the environment throughout their entire lifecycle from cradle to gate compared to traditional textiles due to their complex composition. Therefore, sustainability should be taken into consideration in the development of textiles in addition to their performance. This study employs life cycle assessment to evaluate the system boundary from cradle to gate and analyzes the impact of different stages on the environment. It mainly assesses the impact of textiles on global warming potential, terrestrial acidification potential, freshwater eutrophication potential, freshwater ecotoxicity potential, fossil resource scarcity, and water consumption. The results show that the manufacturing stage is the primary source of environmental impacts which the contribution rate is more than 90%. The main reason is attributed to electricity consumption. Sensitivity analysis indicates that materials such as boron nitride have a greater impact on GWP than other inputs. Establishing regulatory frameworks for nanomaterials and transitioning to clean energy are essential for ensuring that textile innovation delivers both functional and environmental benefits.

**Supplementary Information:**

The online version contains supplementary material available at 10.1007/s11356-026-37632-z.

## Introduction

Textiles are the key interface in direct contact with the human body, and their environmental footprint has become one of the key challenges for global sustainable development (Abagnato et al. 2024; Sun et al. 2024). Studies have shown that the global textile industry contributes approximately 5–10% of greenhouse gas (GHG) emissions and ranks second in terms of water resource consumption (Nørup et al. 2018; Zhang et al. 2023a). The residual dyes and ultra-fine fibers released during the production process, as well as the discarded textiles, significantly increase the risk of aquatic ecotoxicity and may lead to aquatic eutrophication (Jiao et al. 2024). At the same time, with the emergence of harsh environmental conditions, the market demand for textiles with multifunctional properties (such as thermal management, sensing, protection) is growing significantly (Zhang et al. 2023b).

To meet this growing demand for advanced functionality, the field of smart textiles is evolving rapidly. Currently, the development of smart textiles is demonstrating a trend of deep integration among multifunctional unification, interfacial engineering innovation, and structural biomimetic design (Zhu et al. 2025). Low-dimensional nanomaterials such as MXene, CuNWs, and AgNWs are combined with fiber substrates through interfacial engineering techniques like wet spinning, spraying, and dipping. This forms heterogeneously interlocked core-sheath or three-dimensional network structures, integrating various functions including conductivity, sensing, electrothermal/photothermal conversion, and even energy storage (Hou et al. 2024; Fan et al. 2024; He et al. 2024). Meanwhile, interfacial engineering approaches—such as polydopamine functionalization and conductive polymer modification—significantly enhance the adhesion strength and environmental stability between nanomaterials and substrates (Wu et al. 2022; He et al. 2023). This improves the reliability of textiles in practical scenarios such as bending and washing. Furthermore, innovative designs ranging from fabric weaving structures to biomimetic microstructures—like vertically aligned channels and concave light-trapping structures—are advancing the development of smart textiles toward high-performance applications. These include directional transport, photothermal conversion, and adaptive thermal management.

While these advanced functionalities highlight the remarkable progress in smart textiles, their realization relies heavily on synthetic fibers and chemical-intensive processing techniques. For example, Meng et al. (2025) employed electrospinning, chemical polymerization, and electro-spraying techniques with materials such as poly(vinylidene fluoride-co-hexafluoropropylene), polypyrene, polyacrylonitrile, silica, and polydimethylsiloxane to prepare nanofiber textiles with a sandwich structure for outdoor personal thermal management, achieving switchable radiative cooling, solar heating, directional moisture conduction, and self-cleaning functions. Such reliance on synthetic materials and chemical processes not only increases resource consumption and pollutant emissions during production but also significantly prolongs the environmental impact of textiles after disposal in natural environments (Keller et al. 2013; Thomas et al. 2011; Roy Choudhury 2014; Stanton et al. 2023). Therefore, the entire life cycle of textiles, from raw material acquisition and manufacturing to post-use disposal, puts significant pressure on global resource consumption and environmental degradation and poses a severe challenge to the sustainable development goals (SDGs) of China and other countries around the world (Li et al. 2021; Herrera Almanza and Corona 2020).

To systematically reduce the environmental burden of new multifunctional fabrics and identify potential ways to maximize their environmental benefits, the importance of life cycle assessment (LCA) in environmental management and sustainable decision-making for the textile industry has been growing (Wu et al. 2025). LCA can comprehensively quantify resource consumption, energy use, and various environmental impacts related to the entire product lifecycle “from cradle to grave” and conduct objective comparisons among different product systems (Hellweg et al. 2023). There has been extensive application of LCA in textile and clothing, a resource-intensive and environmentally significant industry. A large number of studies focus on analyzing the environmental footprints of different textile raw materials from natural fibers (such as cotton (La Rosa and Grammatikos 2019), silk (Wu et al. 2025), hemp (Van der Werf and Turunen 2008), wool (Wiedemann et al. 2019)) to regenerated cellulose fibers (such as viscose, lyocell, modal) (Shen et al. 2010), and various synthetic fibers (such as polyester (Van der Velden et al. 2014), nylon (Van der Velden et al. 2014), acrylic (Yacout et al. 2016)). These studies reveal the degrees of dependence of different fibers on environmental resources such as water, arable land, and fossil fuels, and their corresponding emission characteristics. At the same time, LCA research also extends to specific textile and clothing products such as bed sheets (De Saxce et al. 2012), towels (Blackburn and Payne 2004), carpets (Sim and Prabhu 2018), and shirts (Kazan et al. 2020; Wiedemann et al. 2020), analyzing the environmental impact hotspots throughout the raw material-to-waste disposal chain and providing data support for product eco-design, material substitution, and process improvement.

However, although the academic community has made significant progress in LCA studies of traditional textiles and single-material products, a notable research gap still exists. There is still limited research on the environmental impact assessment of new high-performance textiles with functions such as radiation heat management (Chen et al. 2025; Zhao et al. 2024), sensing (Yang et al. 2025b), energy harvesting (Yang et al. 2025a), self-cleaning (Zhao et al. 2025), and antibacterial (Hu et al. 2025b). These emerging textiles often achieve their multifunctionality by introducing nanomaterials, functional coatings, composite structures, or intelligent responsive elements. Their manufacturing processes, material composition, and waste disposal characteristics differ significantly from those of traditional textiles (Keller et al. 2013; Shi et al. 2021, 2023; Skrzetuska and Rzeźniczak 2025). This complexity poses new challenges for LCA studies. It requires researchers to model the synthesis and processing of new materials more precisely in order to assess potential new emissions (such as the release of nanoparticles). At the same time, the impact of longer service life or different maintenance requirements on the results should be considered, and the unique environmental behaviors and long-term destinations of their complex components in different waste disposal scenarios (such as landfilling, incineration, or recycling) should be explored (Luján-Ornelas et al. 2020; Sun et al. 2021, 2024). Therefore, systematically conducting LCA studies on these new multifunctional integrated textiles is crucial for accurately quantifying their real environmental performance as a key scientific basis for identifying their potential environmental advantages (or disadvantages) relative to traditional products, optimizing their design, and guiding the textile industry towards a more sustainable direction (Zhu and Liu 2025).

To fill the research gap mentioned above and provide decision support for the development of the next generation of “green” intelligent textiles, this study conducts a cradle-to-gate LCA of laboratory-developed switchable radiation heat-management textiles and benchmarks their environmental impacts against existing literature. By systematically quantifying the environmental footprint across the production chain, the study highlights the main drivers of impact and the potential for improvement. The findings provide evidence to guide the adoption of bio-based alternatives and green synthesis processes, offering practical insights for ecodesign that balances thermal performance with sustainability.

## Methodology and data

**Fig. 1:**
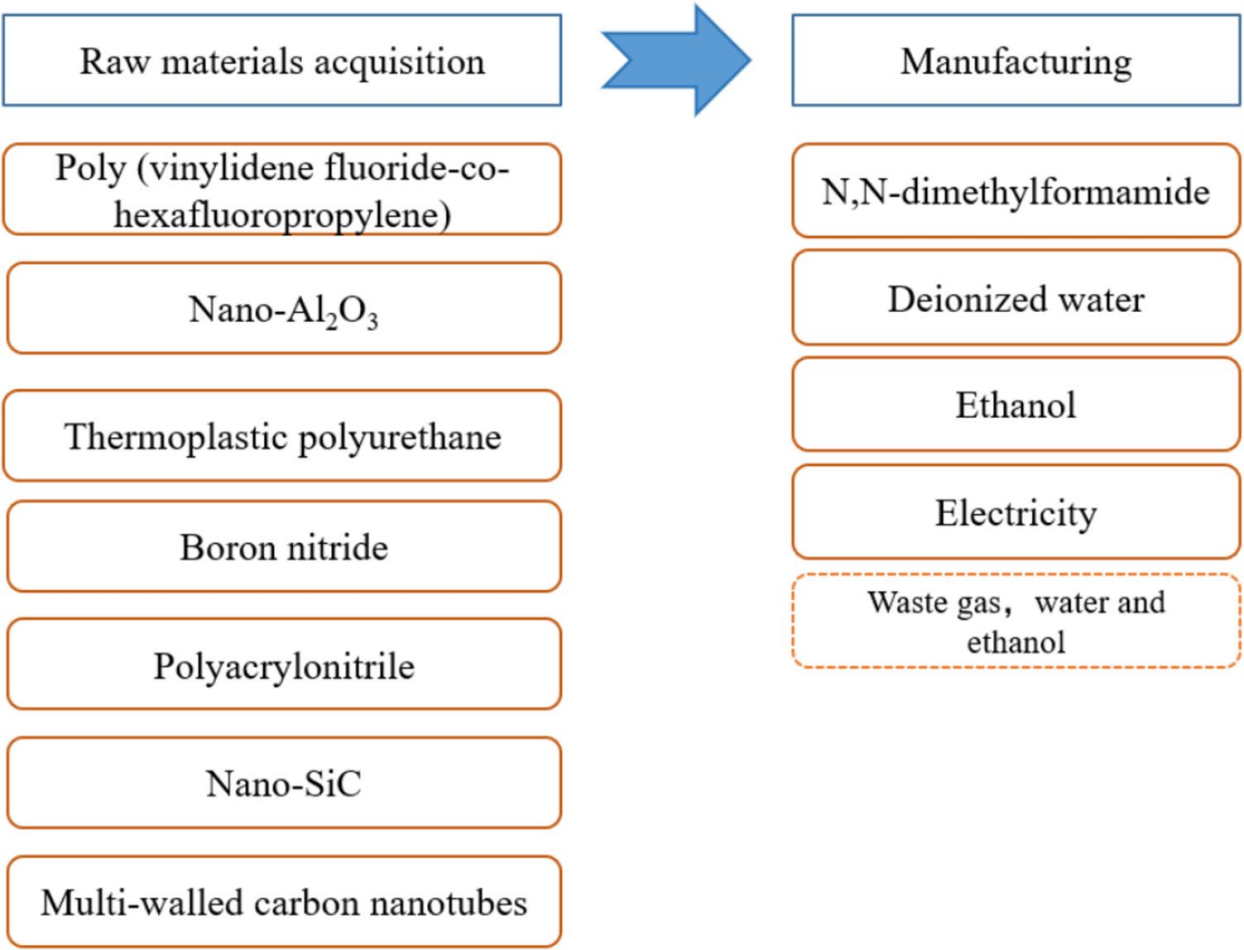
An overview of the system boundary with the sharp-cornered rectangle showing the process flow, the solid-bordered rounded rectangle showing the inputs and the dashed-bordered rounded rectangle showing the outputs

The goal of this LCA is to evaluate the environmental impacts of a novel dual-functional textile from cradle to gate, based on data from current laboratory preparations. This novel textile is divided into radiant cooling side and radiant heating side. Its radiative thermal management functionality is mainly enabled through the application of raw materials such as poly (vinylidene fluoride-co-hexafluoropropylene) (PVDF-HFP), thermoplastic polyurethane (TPU), alumina (Al₂O₃), boron nitride (BN), polyacrylonitrile (PAN), multi-walled carbon nanotubes (MWCNT), and silicon carbide (SiC). The cooling side consists of a matrix based on PVDF-HFP and TPU, incorporating Al₂O₃ and BN as functional particles. The heating side consists of a matrix based on PAN, incorporating MWCNT and SiC as functional particles. Its preparation method is described in detail in the supplementary information. In this study, the system boundaries cover the key stages of new textiles, including the raw materials acquisition stage and the manufacturing stage. And a piece of functional textile (29.5 g) is defined as a functional unit. The study focused on the environmental impact of products in Shanghai, China. And the system boundaries studied by the model are shown in Fig. 1.

The foreground life cycle inventory (LCI) data is based on the actual data used in the preparation of the experiment, including the dosage of reagents used, electricity consumption, consumption of deionized water, and consumption of ethanol. The processing of the foreground data is shown in tables S1-S3 in the supplementary information, and Table 1 shows the inputs and outputs for 29.5 g textile in this study. Modelling was done in SimaPro 9.5. The background LCI database used is Ecoinvent 3.0 (Weidema et al. 2013). If a specific dataset is not available, a Rest of China, Global (GLO), or Rest of the World (RoW) dataset would be used (see table S4).

**Table 1:** Inputs and outputs for producing a piece of textile in this study

Type	Name	Amount	Unit	Data sources
Inputs	PVDF-HFP	6	g	PVDF in Ecoinvent 3.0 (Hu et al. 2023)
	Al_2_O_3_	2	g	Ecoinvent 3.0
	TPU	10	g	Ecoinvent 3.0
	BN	4	g	Data from (Lawal Usman et al. 2022)
	PAN	5	g	Ecoinvent 3.0
	SiC	2	g	Ecoinvent 3.0
	MWCNT	0.5	g	Data from (Temizel-Sekeryan et al. 2021)
	DMF	152	g	Ecoinvent 3.0
	Ethanol	100	g	Ecoinvent 3.0
	Deionized water	100	g	Ecoinvent 3.0
	Electricity	111.3	kWh	Ecoinvent 3.0
Outputs	DMF	152	g	Ecoinvent 3.0
	Waste water	100	mL	Ecoinvent 3.0
	Waste ethanol	100	g	Ecoinvent 3.0

In the raw materials acquisition stage, all data except BN is used directly without any processing. Since the LCI of PVDF-HFP, BN, and MWCNT is not available in the above database, their LCI uses other substances as substitutes or data reported in the literature as data sources. The approach for each material is detailed below, with consistent consideration of data representativeness and methodological precedents.

For PVDF-HFP, data from its fluoropolymer relative, polyvinylidene fluoride (PVDF), can serve as a suitable proxy. This approach is justified because PVDF is a precursor in the synthesis of PVDF-HFP. According to data from Shanghai Chunyi New Material Technology Co., Ltd. (www.sellpa6.com), PVDF-HFP is synthesized via free-radical copolymerization of vinylidene fluoride (VDF) and hexafluoropropylene (HFP) at temperatures ranging from 50 to 80 °C. In this process, VDF typically constitutes a larger molar fraction than HFP. Therefore, PVDF data are often used as a representative model for the PVDF-HFP copolymer system. Moreover, this methodology aligns with established practices in the field. For example, in their evaluation of electrospun nanofiber membranes for membrane distillation, Hu et al. (2023) utilized PVDF data to approximate the properties of PVDF-HFP membranes due to the limited availability of specific copolymer data.

For BN and MWCNT, the available data are mainly based on laboratory-scale studies. There are many articles on LCA analysis and preparation involving BN and MWCNT (Pan et al. 2020; Upadhyayula et al. 2012; Griffiths et al. 2013). We evaluated the technical representativeness of the candidate list (whether conventional industrial preparation methods such as chemical vapor deposition were used for BN and MWCNT), the shape of the materials (for example, the BN used in this study is hexagonal boron nitride nano-powder, and the MWCNT is multi-walled nanostructured powder), and the completeness of the data sources. We ultimately selected the list that provided the most detailed process descriptions and the most transparent data (Lawal Usman et al. 2022; Temizel-Sekeryan et al. 2021).

The inventory data for BN are derived from the data reported by Lawal Usman et al. (2022), as shown in tables S2. MWCNTs are derived from the data reported by Temizel-Sekeryan et al. (2021), as shown in table S3. The data of BN and MWCNT are calculated or referred to in strict accordance with the preparation process of previous studies. The loss of material during preparation is not considered. MWCNT can directly reference the author’s lifecycle inventory data. BN, on the other hand, requires data processing. The provided urea and boric acid data were used to calculate the BN yield by equation CO(NH_2_)_2_ + 2H_3_BO_3_≜2BN + CO_2_ + 5H_2_O, regardless of intermediate process losses. The amount of electricity consumed to prepare 1 kg of BN is based on the experimental data from Abdurakhmonov et al. (2025).

In the manufacturing stage, material loss is not considered during the spinning preparation process. In addition, water consumption is not considered when cleaning the equipment and does not consider the loss of the intermediate production process. Therefore, it can be assumed that the water and ethanol consumption in the list is exclusively used for cleaning materials. For N,N-dimethylformamide (DMF) gas emitted during the production process, it is considered to be directly discharged into the atmosphere. At this stage, the pollutant emissions are wastewater, waste ethanol, and DMF. And the electricity consumed to produce a 25.9 g fabric is shown in table S1 in supplementary information.

The ReCiPe 2016 Midpoint (H)–world (2010) H methodology is employed to quantify the environmental impacts of the textile. This approach represents a benchmark impact assessment method in LCA practice, as evidenced by its extensive adoption in peer-reviewed studies and ISO 14044-compliant assessments (Gonzalez et al. 2023; Hu et al. 2023; Goedkoop et al. 2022; Van Zelm et al. 2025). The method evaluates the degree of impact on the environment by analyzing the following 18 midpoint impact categories: global warming potential (GWP), stratospheric ozone depletion (SOD), ionizing radiation (IR), ozone formation-human health (OF-HH), fine particulate matter formation (FPMF), ozone formation-terrestrial ecosystems (OF-TE), terrestrial acidification potential (TAP), freshwater eutrophication potential (FEP), marine eutrophication potential (MEP), freshwater ecotoxicity potential (FEP-1), marine ecotoxicity potential (MEP-1), terrestrial ecotoxicity potential (TEP), human carcinogenic toxicity potential (HCTP), human non-carcinogenic toxicity potential (HNCTP), land use (LU), mineral resource scarcity (MRS), fossil resource scarcity (FRS), and water consumption (WC).

The 18 impact categories are used to quantify the impact of the fabric on climate change, energy consumption, and damage to people and the natural environment (Xu et al. 2021). Greenhouse gas emissions and fossil energy consumption are the most concerned environmental issues in China in recent years, followed by acidification, eutrophication, toxic effects, and water consumption (Sandin and Peters 2018; Meys et al. 2021). Among the aforementioned 18 impact categories, MEP, MEP-1, HCTP, and HNCTP are classified as categories that are not frequently assessed (Zhang et al. 2015; Muthu et al. 2012; Morita et al. 2020; Baydar et al. 2015; Fidan et al. 2023). Therefore, this study focuses on the contribution of novel textiles to GWP, TAP, FEP, FEP-1, FRS, and WC to evaluate their environmental impact in China. Table 2 provides a detailed explanation of them. For explanations of the remaining impact categories, please see table S5 in the supplementary information.

**Table 2:** Explanation of the six selected impact categories

Impact category	Abbreviation	Definition
Global warming potential	GWP	Contribution of greenhouse gas emissions to climate change through heat trapping in the atmosphere
Terrestrial acidification potential	TAP	Emissions that increase soil and terrestrial ecosystem acidity
Freshwater eutrophication potential	FEP	Nutrient-driven enrichment of freshwater systems, leading to algal growth and oxygen depletion
Freshwater ecotoxicity potential	FEP-1	Potential toxic effects of chemical emissions on freshwater organisms
Fossil resource scarcity	FRS	Depletion of fossil fuel resources and long-term availability constraints
Water consumption	WC	Net consumption of freshwater resources within a watershed

We conducted a sensitivity analysis to further evaluate the environmental contribution of each textile raw material. By varying material content across six levels (100 to 0%), the analysis identifies those with the highest impact on environmental performance. The results provide empirical evidence to inform sustainable material selection and support ecological design in engineering practice.

## Results

**Fig. 2:**
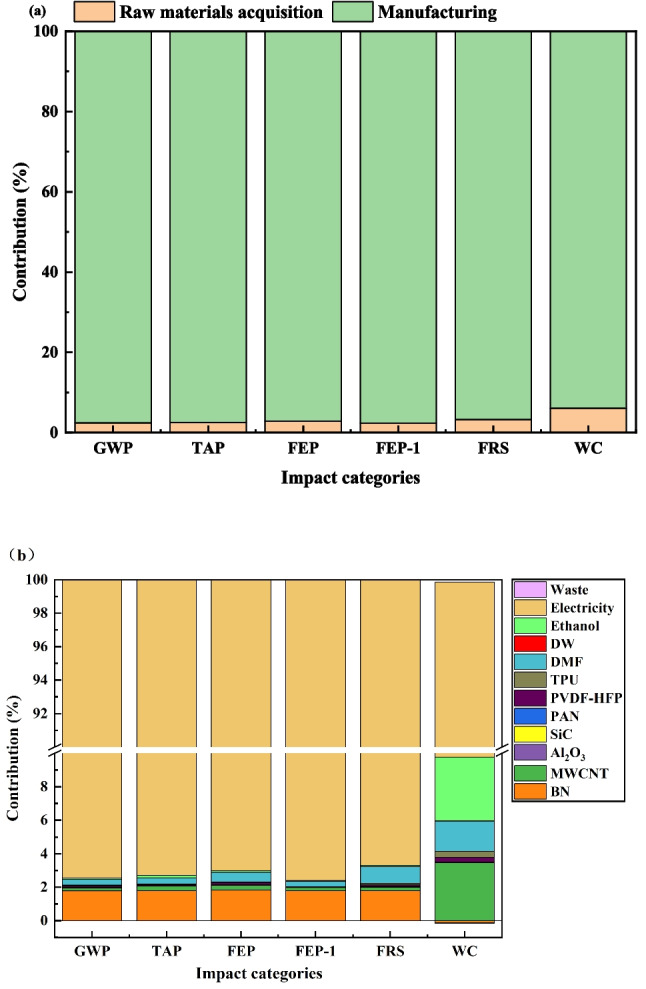
**a** The life cycle environmental footprint of new textiles in China and **b** the main contribution of inputs to the category. DW, DMF, TPU, PVDF-HFP, PAN, and MWCNT are the abbreviations for deionized water, N,N-dimethylformamide, thermoplastic polyurethane, poly (vinylidene fluoride-co-hexafluoropropylene), polyacrylonitrile, and multi-walled carbon nanotubes. And Al₂O₃, BN, and SiC are the chemical formulas for alumina, boron nitride, and silicon carbide. Waste DMF (gas), wastewater, and waste ethanol are included in waste

The environmental footprint (see table S6 for detailed values) and primary contributions across the life cycle of the textile are presented in Fig. 2a and b, respectively. As summarized in table S6, the production of a single textile piece generates a GWP of 125.5 kg CO₂ eq. Other significant environmental impacts include a TAP of 434.2 g SO₂ eq, a FEP of 24.9 g P eq, a FEP-1 of 3.99 kg 1,4-DCB, an FRS of 24.6 kg oil eq, and a WC of 324.4 L. As shown in Fig. 2a, the manufacturing stage is the dominant contributor, accounting for over 93% of the impacts in these categories. This dominance is primarily attributed to electricity consumption, which, as revealed in Fig. 2b, is responsible for more than 90% of the contributions within the manufacturing stage across these impact categories.

The high electricity demand stems from specific production processes: electrospinning (66 kWh) and vacuum drying (44.1 kWh) for a piece of textile. This central role of manufacturing-stage electricity underscores its greater environmental impact compared to the raw materials acquisition stage.

Building on the finding that manufacturing-stage electricity consumption is the primary environmental hotspot, this sensitivity analysis further examines the role of individual materials added during the raw materials acquisition stage. Although these components enhance the textile’s thermal management, their production carries distinct environmental burdens.

**Fig. 3:**
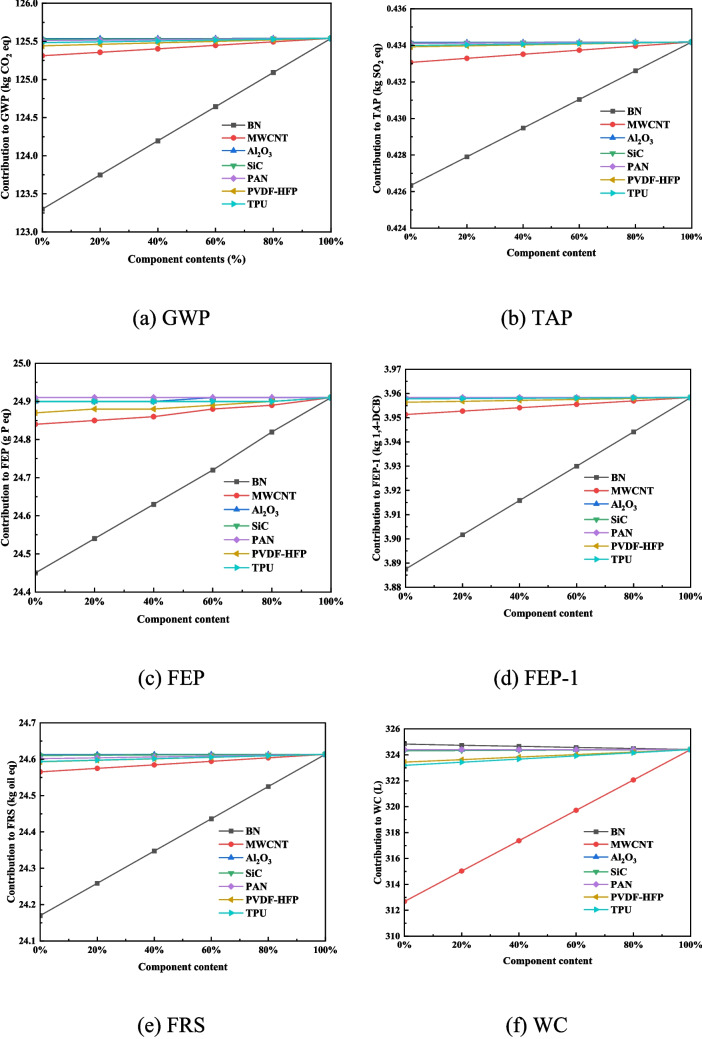
The sensitivity analysis of different components

As shown in Fig. 3, reducing the BN content brings the greatest marginal benefit to GWP, lowering it from 125.5 kg CO₂ eq to 123.3 kg CO₂ eq. At the same time, it also leads to the largest decreases in TAP (8 g SO₂ eq), FEP (0.5 g P eq), FEP-1 (70 g 1,4-DCB), and FRS (443.7 g oil eq). In contrast, reducing the MWCNT content has the most significant impact on WC, decreasing it by 11.7 L, which is an order of magnitude greater than the effect of reducing other polymers. Overall, BN is the dominant contributor of GWP, TAP, FEP, FEP-1, and FRS, while MWCNT is the primary driver for WC. Results for the remaining categories are reported in Table S2 in the Supplementary Information.

This disproportionate influence of BN and MWCNT is directly linked to their disproportionately high embodied energy. According to reports by Abdurakhmonov et al. (2025) and Mauron et al. (2003), the production of 1 kg of BN requires 400–600 kWh, and 1 kg of MWCNT requires 174 kWh (Table S7), vastly exceeding the energy required for other constituents. Consequently, targeting these two materials for substitution with functionally equivalent but less energy-intensive alternatives presents the most effective pathway for reducing the cradle-to-gate environmental impact of this textile.

To clarify the environmental performance of the textile studied, a comparative analysis with Wu et al. (2025) was conducted. Wu’s work evaluates the environmental impact of producing 1 kg of raw silk in China using a cradle-to-gate system boundary. Silk was selected as a benchmark for this comparison because it represents a traditional, natural, high-performance fiber with well-documented LCA studies in the Chinese context (Liu et al. 2022). Presently, no published LCA studies are available for textiles with performance characteristics comparable to the products analyzed in this study. Silk serves as the most suitable reference for contextualizing our results, offering a meaningful point of reference for assessing potential advantages and trade-offs. Both studies apply the same methodology, ReCiPe 2016 Midpoint (H). Since the functional units differ—1 kg of raw silk in Wu’s study versus 29.5 g of fabric in this work—the data from Wu et al. were normalized by a factor of 0.0295 to enable a consistent comparison. Detailed results are available in tables S8 and S9 of the supplementary information.

The environmental profiles of the two studies diverge significantly in their primary influencing stages. For this study, the manufacturing stage exerts the most substantial influence, a consequence of the high electricity demand from electrospinning operations like spinning and drying. The scale of this consumption is highlighted by the 111.3 kWh required to produce only 29.5 g of fabric. Although electricity is also the main contributor within the raw materials acquisition stage—attributed to the energy-intensive synthesis of BN and MWCNT—its cumulative contribution to the overall environmental footprint remains minor (< 6.1%). This stands in stark contrast to the system boundary defined by Wu et al., where the agricultural (mulberry planting) stage is predominant. In that context, urea use is the critical driver, accounting for more than 59% of the impacts from materials. The significance of urea is further corroborated by a sensitivity analysis, which projected that a mere 5% reduction in its application would yield environmental benefit equivalents of 4.5% in terrestrial ecotoxicity and 4.1% in freshwater ecotoxicity. In the subsequent sericulture and silk reeling stages of Wu’s study, energy inputs in the form of electricity and steam again become the leading factors.

## Discussions

In this study, the environmental impact of a piece of textile in China was systematically evaluated through LCA, and the analysis results clearly revealed the key hotspots of its environmental footprint. Through the above analysis, it is found that electricity, BN, and MWCNT are the main influencing factors, which need further improvement. These findings have important implications for formulating relevant policies, guiding industrial transformation, and planning future research.

LCA identifies electricity consumption as the principal factor driving the environmental impacts of the new textile across its life cycle (Fig. 2). This is largely attributable to the high energy demands of electrospinning and vacuum drying, consistent with observations by Hu et al. (2023). Their study noted that electrospinning operates at high voltages for prolonged periods (≥ 3 h), leading to considerable electricity use. In the case of E-PH membranes, this process accounted for approximately 72% of the total midpoint environmental impacts.

The substantial electricity consumption associated with electrospinning stems from the sustained application of a high voltage differential—17 kV in this study—over prolonged processing periods (≥ 44 h), a process inherently subject to Joule heating and other energy losses. While this high-voltage requirement is intrinsic to the technique, its operational efficiency could be enhanced; for instance, utilizing a multi-needle setup could significantly reduce total processing time. Furthermore, as illustrated in Fig. 2, the resulting environmental burden is directly linked to the fossil fuel–dominated electricity grid, which generates emissions of CO₂, SO₂, and other combustion by-products (Liu et al. 2007).

Consequently, integrating electricity from clean energy sources represents a viable strategy to mitigate these impacts (Messagie et al. 2014). Realizing this transition will require policy-driven initiatives, including financial incentives and technical support, to facilitate the adoption of renewables and energy-saving innovations. These efforts are essential to steer the textile industry toward a more sustainable and competitive future.

Sensitivity analysis within the LCA framework further reveals that MWCNT and BN are the most environmentally consequential materials in the textile production inventory. Their influence is tied directly to the high embodied energy from synthesis. For MWCNT, the primary source of impact is electricity consumption during chemical vapor deposition (CVD)—a process that necessitates sustained high temperatures (typically exceeding 600 °C) and a vacuum to facilitate gas-phase precursor reactions and nanoscale deposition (Temizel-Sekeryan et al. 2021). This conclusion is consistent with the findings of Bauer et al. (2008), who noted that the impact is almost exclusively due to the CVD process itself.

While comprehensive LCAs for BN are scarce, its synthesis pathways share a reliance on high-temperature and high-pressure conditions, suggesting a similarly significant energy footprint. This observation aligns with broader LCA literature, which identifies energy-intensive materials as principal drivers of environmental loading in systems such as batteries (Carlin et al. 2024). A critical paradox emerges: although MWCNT and BN are incorporated to enhance photothermal performance (MWCNT boosts absorption, while BN improves reflectivity), their substantial embodied energy burden risks negating the operational energy savings achieved during the use stage (e.g., reduced air conditioning demand).

To reconcile functional performance with sustainability, a shift toward bio-based or low-energy functional materials is advised. Promising alternatives include UV-reflective mulberry extracts or silk fibroin derivatives as partial substitutes for BN (Ghosh et al. 2025). And although the environmental profile of graphene oxide requires further optimization, its aqueous synthesis route presents a lower-energy alternative to CVD for MWCNT (Zhao et al. 2012). Furthermore, adopting integrated, renewable-powered production systems—such as solar-powered CVD, inspired by integrated water-fertilizer practices in silk production (Wang and Zhang 2021)—could decouple material synthesis from grid-based fossil energy.

In general, using electricity generated from clean energy and bio-based materials is an effective way to enhance the environmental benefits of new fabrics from raw material acquisition to the production process. In addition, electrospinning technology can be used to alter the structure of fabrics to improve thermal management performance of the fabrics (Shepa et al. 2021). According to Mie scattering theory, researchers can regulate the optical properties of fabrics during the electrospinning process by controlling the fiber diameter through adjusting the solution concentration, voltage intensity, and nozzle size (Liu et al. 2024; Blachowicz and Ehrmann 2023). As reported by Pyun et al. (2024), by precisely controlling the introduction of micro- and nanoscale fiber structures (0.3–2.5 microns) through electrospinning technology, it is possible to efficiently induce strong sunlight scattering, thereby achieving a high solar reflectance (93%). This technology can be used not only in the preparation of high-performance fabrics but also to improve the thermal management performance of traditional fabrics. Beyond enabling high-performance functional fabrics, such structural optimization can also be applied to conventional textiles, offering a pathway to reduce reliance on hazardous functional additives such as BN and MWCNT and thereby lower the overall environmental impacts.

This study offers valuable insights into the environmental impacts of new textile production; however, several limitations must be acknowledged. These primarily stem from two overarching sources: the gap between laboratory-scale experiments and industrial production and the absence of localized data. The following analysis details these limitations and outlines essential directions for future research.

The discrepancies between laboratory-scale preparation and industrial-scale production can be categorized into three aspects: (1) Data for PVDF-HFP synthesis were derived from the material inventory of its primary precursor, PVDF. (2) Information regarding BN and MWCNT was obtained from existing literature, which may not fully represent the conditions of this study. (3) The energy efficiency and environmental impacts estimated for the manufacturing stage may not accurately reflect actual industrial performance. Each of these aspects will be discussed in detail below.

First, the LCA for the copolymer PVDF-HFP relied on inventory data for its precursor, PVDF. Using a PVDF inventory likely underestimates the environmental impact, as synthesizing PVDF-HFP is a more complex process. It involves additional materials such as initiators (benzoyl peroxide) and solvents (DMF) and entails post-synthesis steps like precipitation, washing, and drying—all contributing extra energy and material burdens not captured by PVDF data alone.

Second, the data for BN and MWCNT were sourced from the literature, which may not reflect scale-specific efficiencies. While lab methods mirror industrial processes, actual production operates at a far larger scale with superior resource efficiency. For example, in large-scale MWCNT production, Gavankar et al. (2015) report substantial efficiency gains per unit of material produced: reductions of 96% in feedstock, 62% in carrier gas, 96% in hydrogen, 50% in catalysts and deionized water, and 87% in electricity usage. While emission proportions per unit output are often assumed similar across scales, electricity consumption remains the primary driver of environmental impact in both BN and MWCNT synthesis. Furthermore, the regional power generation mix significantly influences LCA outcomes. As noted by Temizel-Sekeryan et al. (2021), shifting from grid electricity to nuclear power for the same material could reduce emissions by 5.86 × 10^5^ kg CO₂ eq/kg, whereas using coal-based power would increase emissions by 2.05 × 10^5^ kg CO₂ eq/kg.

The third relates to energy efficiency and overall environmental performance at the experimental production scale. The electrospinning equipment used in this study is a small-scale laboratory setup, which exhibits lower production efficiency and higher energy consumption per unit output compared to industrial production lines. For instance, data from Shenzhen Tongli Micro‑Nano Technology Co., Ltd. (www.tlwnt.com) indicate that producing 1 kg of fabric at mass‑production scale consumes approximately 20 kWh of electricity. Similarly, mass‑production data from Shandong Nafibo Technology Development Co., Ltd. (www.nafiber.com.cn) show that generating 200 g of fiber requires about 55 kWh. Moreover, industrial production typically employs multi‑nozzle synchronous spinning and integrates waste‑liquid and waste‑gas recovery systems. These features not only shorten spinning time but also significantly reduce waste emissions, further enhancing the environmental profile of full‑scale manufacturing.

The absence of localized data for certain materials constitutes another key limitation of this study. Most material inventories used in this paper are from Global (GLO) or Rest of the World (RoW) (see Table S4 in the supplementary information for details), which may not accurately reflect conditions in China. Hu et al. (2025a) conducted a comparative life cycle analysis between the international Ecoinvent database and Chinese local databases, revealing systematic biases in the carbon accounting of power equipment. Their findings indicate that, per equivalent functional unit, the Chinese local database yields result 6413.68 kg CO₂ eq higher than the Ecoinvent database, representing an absolute deviation rate of 19.97%. This implies that the results of this study are comparatively conservative.

This study does not encompass the product’s use and end-of-life stages. In a study of cotton T‑shirts, Baydar et al. (2015) find that the use stage contributes approximately 47.7% to the GWP over the entire life cycle (from cotton cultivation and harvesting to the disposal of T-shirts). The disposal stage (considering only incineration) accounts for about 5.9%. Their analysis assumes an energy consumption of around 50 kWh during the use stage (wear and washing) for a cotton T‑shirt. In contrast, the products examined in this study are expected to reduce users’ reliance on air conditioning, fans, and other temperature‑control devices due to their radiant heat-management function, thereby offsetting a portion of energy use in the use stage. Consequently, the global warming impact of these products during the use stage should be lower than that of cotton T‑shirts. However, in the disposal stage, given that the product incorporates multiple chemical materials, the potential environmental hazards arising from incineration are anticipated to be substantially higher than those of purely natural cotton products. Furthermore, studies for recycling and reuse remain unexplored in the current literature. Therefore, a full “cradle-to-cradle” model is essential to inform the industry on eco-design strategies.

To overcome these limitations, future work should focus on developing robust, localized life cycle inventories through primary data collection, alongside comprehensive cradle-to-cradle modelling. Given the early-stage development of the products and the absence of robust socio-economic data, future work should pursue prospective or scenario-based extensions of LCA that integrate indicative economic and technical parameters to inform early-stage design and scaling decisions.

In conclusion, while this study provides a preliminary assessment, its findings highlight the need for more granular, localized data and expanded system boundaries. Addressing these gaps through the outlined research directions will enable a more robust and actionable evaluation of the sustainable potential of advanced textile technologies.

## Conclusion

A cradle-to-gate LCA was conducted to evaluate the environmental performance of a novel lab-made textile in China. We evaluated its contributions to 18 impact categories and identified the primary environmental hotspots within the system.

The results demonstrate that the textile’s environmental footprint is predominantly concentrated in the manufacturing stage, which accounts for over 90% of the impacts in key categories including global warming potential (GWP), terrestrial acidification potential (TAP), freshwater eutrophication potential (FEP), freshwater ecotoxicity potential (FEP-1), fossil resource scarcity (FRS), and water consumption (WC). Over its life cycle, the textile was responsible for 125.5 kg CO₂ eq, 434.2 g SO₂ eq, 24.9 g P eq, 3.9 kg 1,4-DCB, 24.6 kg oil eq, and 324.4 L. Material sensitivity analysis identified boron nitride (BN) and multi-walled carbon nanotubes (MWCNT) as the principal drivers of environmental impact during the raw materials. A comparative analysis with existing LCA studies on silk production in China revealed that the novel textile exerts 51.3%, 23.1%, 83.4%, 99%, 40%, and 2% greater environmental burdens in the aforementioned categories, respectively. The primary underlying cause for these results is attributed to the reliance on fossil fuel–based electricity.

Our findings help researchers better understand the importance of establishing systematically optimized strategies to reduce the environmental impact of fabrics. Therefore, researchers and related practitioners are encouraged to further strengthen the study of China’s new material production processes and energy structure in the future, in order to provide more localized data and decision support. At the same time, the life cycle inventory and results of this study can offer some insights for the sustainable development of new functional textiles in China.

## Supplementary Information

Below is the link to the electronic supplementary material.

ESM 1 (DOCX 521 KB)

## Data Availability

The data that support the findings of this study are available from the corresponding author upon reasonable request.

## References

[CR1] Abagnato S, Rigamonti L, Grosso M (2024) Life cycle assessment applications to reuse, recycling and circular practices for textiles: a review. Waste Manag 182:74–90 38643525 10.1016/j.wasman.2024.04.016

[CR2] Abdurakhmonov O, Aripova M, Erkinov F, Abdurakhmonov S, Sharopov U, Karimov M, Kurbanov M, Saidov D, Pędzich Z, Kozien D, Kurniawan TA, Bondar E (2025) Green synthesis of high-purity hexagonal boron nitride nanoparticles. Vacuum 239:114386

[CR3] Bauer C, Buchgeister J, Hischier R, Poganietz W-R, Schebek L, Warsen J (2008) Towards a framework for life cycle thinking in the assessment of nanotechnology. J Clean Prod 16:910–926

[CR4] Baydar G, Ciliz N, Mammadov A (2015) Life cycle assessment of cotton textile products in Turkey. Resour Conserv Recycl 104:213–223

[CR5] Blachowicz T, Ehrmann A (2023) Optical properties of electrospun nanofiber mats. Membranes (Basel) 13:441 37103868 10.3390/membranes13040441 PMC10146296

[CR6] Blackburn RS, Payne JD (2004) Life cycle analysis of cotton towels: impact of domestic laundering and recommendations for extending periods between washing. Green Chem 6:G59–G61

[CR7] Carlin M, Kaur J, Ciobanu DZ, Song Z, Olsson M, Totu T, Gupta G, Peng G, González VJ, Janica I (2024) Hazard assessment of hexagonal boron nitride and hexagonal boron nitride reinforced thermoplastic polyurethane composites using human skin and lung cells. J Hazard Mater 473:134686 38788582 10.1016/j.jhazmat.2024.134686

[CR8] Chen Z, Zhang Q, Ding L, Lv G, Liu T, Yang Z, Jiang Y, Li L, Li W, Ding F, Xu W, Zhu J, Zhu B (2025) An infrared-transparent textile with high drawing processed Nylon 6 nanofibers. Nat Commun 16:2009 40011498 10.1038/s41467-025-57366-9 PMC11865603

[CR9] De Saxce M, Pesnel S, Perwuelz A (2012) Lca of bed sheets – some relevant parameters for lifetime assessment. J Clean Prod 37:221–228

[CR10] Fan H, Wang K, Ding Y, Qiang Y, Yang Z, Xu H, Li M, Xu Z, Huang C (2024) Core–shell composite nanofibers with high temperature resistance, hydrophobicity and breathability for efficient daytime passive radiative cooling. Adv Mater. 10.1002/adma.202406987 10.1002/adma.202406987 39194411

[CR11] Fidan FŞ, Aydoğan EK, Uzal N (2023) Recent progress on life cycle sustainability assessment in textile industry: applications for environmental, economic, and social impacts of cotton and its derivatives. In: Muthu SS (ed) Progress on Life Cycle Assessment in Textiles and Clothing. Springer Nature Singapore, Singapore

[CR12] Gavankar S, Suh S, Keller AA (2015) The role of scale and technology maturity in life cycle assessment of emerging technologies: a case study on carbon nanotubes. J Ind Ecol 19:51–60

[CR13] Ghosh J, Repon MR, Kulsum U, Refat JK, Jahan M, Rupanty NS, Asif TR, Reukov V (2025) Multifunctional protein-based fibrous material using ivy gourd extract. Surf Innov 13:1–13

[CR14] Goedkoop M, Oele M, De Schryver A, Vieira M (2022) SimaPro 9.0: database manual methods, v4.15, Database & Support team at PRé Sustainability.

[CR15] Gonzalez V, Lou X, Chi T (2023) Evaluating environmental impact of natural and synthetic fibers: a life cycle assessment approach. Sustainability 15:7670

[CR16] Griffiths OG, O’Byrne JP, Torrente-Murciano L, Jones MD, Mattia D, McManus MC (2013) Identifying the largest environmental life cycle impacts during carbon nanotube synthesis via chemical vapour deposition. J Clean Prod 42:180–189

[CR17] He Y, Liu Q, Tian M, Zhang X, Qu L, Fan T, Miao J (2023) Highly conductive and elastic multi-responsive phase change smart fiber and textile. Compos Commun 44(2023):101772

[CR18] He Y, Guo S, Zuo X, Tian M, Zhang X, Qu L, Miao J (2024) Smart green cotton textiles with hierarchically responsive conductive network for personal healthcare and thermal management. ACS Appl Mater Interfaces 16:59358–59369 39422650 10.1021/acsami.4c13999

[CR19] Hellweg S, Benetto E, Huijbregts MAJ, Verones F, Wood R (2023) Life-cycle assessment to guide solutions for the triple planetary crisis. Nature Reviews Earth & Environment 4:471–486

[CR20] Herrera Almanza AM, Corona B (2020) Using social life cycle assessment to analyze the contribution of products to the Sustainable Development Goals: a case study in the textile sector. Int J Life Cycle Assess 25:1833–1845

[CR21] Hou Z, He Y, Qu L, Zhang X, Fan T, Miao J (2024) Core–sheath heterogenous interlocked stretchable conductive fiber induced by adhesive MXene modulated interfacial soldering. Nano Lett 24:15142–15150 39555726 10.1021/acs.nanolett.4c04731

[CR22] Hu X, Guo J, An AKJ, Chopra SS (2023) Electrospun nanofibrous membranes for membrane distillation application—a dynamic life cycle assessment (dLCA) approach. Water Res 243:120376 37516077 10.1016/j.watres.2023.120376

[CR23] Hu J, Jiang X, Gao F, Liu T, Li Z (2025a) Bias in carbon footprint accounting of GIS: an empirical study based on ecoinvent and Chinese lcal data, IEEE.

[CR24] Hu N, Zhu Z, Cai X, Müller-Buschbaum P, Zhong Q (2025b) Enhanced anti-bacterial properties and thermal regulation via photothermal conversion with localized surface plasmon resonance effect in cotton fabrics. J Colloid Interface Sci 681:25–34 39591852 10.1016/j.jcis.2024.11.138

[CR25] Jiao H, Ali SS, Alsharbaty MHM, Elsamahy T, Abdelkarim E, Schagerl M, Al-Tohamy R, Sun J (2024) A critical review on plastic waste life cycle assessment and management: challenges, research gaps, and future perspectives. Ecotoxicol Environ Saf 271:115942 38218104 10.1016/j.ecoenv.2024.115942

[CR26] Kazan H, Akgul D, Kerc A (2020) Life cycle assessment of cotton woven shirts and alternative manufacturing techniques. Clean Technol Environ Policy 22:849–864

[CR27] Keller AA, McFerran S, Lazareva A, Suh S (2013) Global life cycle releases of engineered nanomaterials. J Nanopart Res 15:1692

[CR28] La Rosa AD, Grammatikos SA (2019) Comparative life cycle assessment of cotton and other natural fibers for textile applications. Fibers 7:101

[CR29] Lawal Usman U, Kumar Allam B, Bahadur Singh N, Banerjee S (2022) Adsorptive removal of Cr(VI) from wastewater by hexagonal boron nitride-magnetite nanocomposites: kinetics, mechanism and LCA analysis. J Mol Liq 354:118833

[CR30] Li X, Wang L, Ding X (2021) Textile supply chain waste management in China. J Clean Prod 289:125147

[CR31] Liu L, Cheng SY, Li JB, Huang YF (2007) Mitigating environmental pollution and impacts from fossil fuels: the role of alternative fuels. Energy Sources Part A Recovery Util Environ Eff 29:1069–1080

[CR32] Liu S, Liu H, Meng Y, Li Q, Wang L (2022) Review of carbon emission and carbon neutrality in the life cycle of silk products. Fibres Text East Eur. 10.2478/ftee-2022-0001

[CR33] Liu R, Wang S, Zhou Z, Zhang K, Wang G, Chen C, Long Y (2024) Materials in radiative cooling technologies. Adv Mater 37:2401577 38497602 10.1002/adma.202401577 PMC11733833

[CR34] Luján-Ornelas C, Güereca LP, Franco-García M-L, Heldeweg M (2020) A life cycle thinking approach to analyse sustainability in the textile industry: a literature review. Sustainability 12:10193

[CR35] Mauron P, Emmenegger C, Sudan P, Wenger P, Rentsch S, Züttel A (2003) Fluidised-bed CVD synthesis of carbon nanotubes on Fe2O3/MgO. Diamond Relat Mater 12:780–785

[CR36] Meng X, Zhang M, Zhao Q, Li Q, Chen X (2025) A sandwich-structured nanofibrous textile with moisture wicking for passive radiative cooling and heating. Chem Eng J 509:161272

[CR37] Messagie M, Mertens J, Oliveira L, Rangaraju S, Sanfelix J, Coosemans T, Van Mierlo J, Macharis C (2014) The hourly life cycle carbon footprint of electricity generation in Belgium, bringing a temporal resolution in life cycle assessment. Appl Energy 134:469–476

[CR38] Meys R, Kätelhön A, Bachmann M, Winter B, Zibunas C, Suh S, Bardow A (2021) Achieving net-zero greenhouse gas emission plastics by a circular carbon economy. Science 374:71–76 34591623 10.1126/science.abg9853

[CR39] Morita AM, Moore CCS, Nogueira AR, Kulay L, Ravagnani MAdSS (2020) Assessment of potential alternatives for improving environmental trouser jeans manufacturing performance in Brazil. J Clean Prod 247:119156

[CR40] Muthu SS, Li Y, Hu JY, Mok PY (2012) Quantification of environmental impact and ecological sustainability for textile fibres. Ecol Indic 13:66–74

[CR41] Nørup N, Pihl K, Damgaard A, Scheutz C (2018) Development and testing of a sorting and quality assessment method for textile waste. Waste Manag 79:8–21 30343814 10.1016/j.wasman.2018.07.008

[CR42] Pan D, Su F, Liu H, Ma Y, Das R, Hu Q, Liu C, Guo Z (2020) The properties and preparation methods of different boron nitride nanostructures and applications of related nanocomposites. Chem Rec 20:1314–1337 32959523 10.1002/tcr.202000079

[CR43] Pyun KR, Jeong S, Yoo MJ, Choi SH, Baik G, Lee M, Song J, Ko SH (2024) Tunable radiative cooling by mechanochromic electrospun micro‐nanofiber matrix. Small 20:2308572 10.1002/smll.202308572 38087885

[CR44] Roy Choudhury AK (2014) Environmental impacts of the textile industry and its assessment through life cycle assessment. In: Muthu SS (ed) Roadmap to sustainable textiles and clothing: environmental and social aspects of textiles and clothing supply chain. Springer Singapore, Singapore

[CR45] Sandin G, Peters GM (2018) Environmental impact of textile reuse and recycling–a review. J Clean Prod 184:353–365

[CR46] Shen L, Worrell E, Patel MK (2010) Environmental impact assessment of man-made cellulose fibres. Resour Conserv Recycl 55:260–274

[CR47] Shepa I, Mudra E, Dusza J (2021) Electrospinning through the prism of time. Mater Today Chem 21:100543

[CR48] Shi X, Zuo Y, Zhai P, Shen J, Yang Y, Gao Z, Liao M, Wu J, Wang J, Xu X (2021) Large-area display textiles integrated with functional systems. Nature 591:240–245 33692559 10.1038/s41586-021-03295-8

[CR49] Shi HH, Pan Y, Xu L, Feng X, Wang W, Potluri P, Hu L, Hasan T, Huang YYS (2023) Sustainable electronic textiles towards scalable commercialization. Nat Mater 22:1294–1303 37500958 10.1038/s41563-023-01615-z

[CR50] Sim J, Prabhu V (2018) The life cycle assessment of energy and carbon emissions on wool and nylon carpets in the United States. J Clean Prod 170:1231–1243

[CR51] Skrzetuska E, Rzeźniczak P (2025) Circularity of smart products and textiles containing flexible electronics: challenges, opportunities, and future directions. Sensors Basel 25:1787 40292923 10.3390/s25061787 PMC11946298

[CR52] Stanton T, Stanes E, Gwinnett C, Lei X, Cauilan-Cureg M, Ramos M, Sallach JB, Harrison E, Osborne A, Sanders CH, Baynes E, Law A, Johnson M, Ryves DB, Sheridan KJ, Blackburn RS, McKay D (2023) Shedding off-the-grid: the role of garment manufacturing and textile care in global microfibre pollution. J Clean Prod 428:139391

[CR53] Sun X, Wang X, Sun F, Tian M, Qu L, Perry P, Owens H, Liu X (2021) Textile waste fiber regeneration via a green chemistry approach: a molecular strategy for sustainable fashion. Adv Mater 33:2105174 10.1002/adma.202105174 34561908

[CR54] Sun G, Cao X, Wang Y, Sun X, Chen Q (2024) Comparative life cycle assessment of two different waste materials for recycled fiber. Resour Conserv Recycl 205:107518

[CR55] Temizel-Sekeryan S, Wu F, Hicks AL (2021) Global scale life cycle environmental impacts of single- and multi-walled carbon nanotube synthesis processes. Int J Life Cycle Assess 26:656–672

[CR56] Thomas CR, George S, Horst AM, Ji Z, Miller RJ, Peralta-Videa JR, Xia T, Pokhrel S, Mädler L, Gardea-Torresdey JL, Holden PA, Keller AA, Lenihan HS, Nel AE, Zink JI (2011) Nanomaterials in the environment: from materials to high-throughput screening to organisms. ACS Nano 5:13–20 21261306 10.1021/nn1034857

[CR57] Upadhyayula VKK, Meyer DE, Curran MA, Gonzalez MA (2012) Life cycle assessment as a tool to enhance the environmental performance of carbon nanotube products: a review. J Clean Prod 26:37–47

[CR58] Van der Werf HMG, Turunen L (2008) The environmental impacts of the production of hemp and flax textile yarn. Ind Crops Prod 27:1–10

[CR59] Van der Velden NM, Patel MK, Vogtländer JG (2014) LCA benchmarking study on textiles made of cotton, polyester, nylon, acryl, or elastane. Int J Life Cycle Assess 19:331–356

[CR60] Van Zelm R, Hennequin T, Huijbregts MAJ (2025) Performing life cycle impact assessment with the midpoint and endpoint method ReCiPe. Nat Protoc. 10.1038/s41596-025-01207-y 10.1038/s41596-025-01207-y 40634596

[CR61] Wang Y, Zhang H, (2021) Application of water and fertilizer integration technology in agricultural production. Fifth International Conference on Traffic Engineering and Transportation System (ICTETS 2021). 12058, 1125–1129.

[CR62] Weidema, B. P., Bauer, C., Hischier, R., Mutel, C., Nemecek, T., Reinhard, Jr., Vadenbo, C. O., Wernet, G., 2013. The ecoinvent database: overview and methodology, Data quality guideline . The ecoinvent database, v3.

[CR63] Wiedemann SG, Simmons A, Watson KJL, Biggs L (2019) Effect of methodological choice on the estimated impacts of wool production and the significance for LCA-based rating systems. Int J Life Cycle Assess 24:848–855

[CR64] Wiedemann SG, Biggs L, Nebel B, Bauch K, Laitala K, Klepp IG, Swan PG, Watson K (2020) Environmental impacts associated with the production, use, and end-of-life of a woollen garment. Int J Life Cycle Assess 25:1486–1499

[CR65] Wu J, Wang M, Dong L, Shi J, Ohyama M, Kohsaka Y, Zhu C, Morikawa H (2022) A trimode thermoregulatory flexible fibrous membrane designed with hierarchical core–sheath fiber structure for wearable personal thermal management. ACS Nano 16:12801–12812 35947793 10.1021/acsnano.2c04971

[CR66] Wu Z, Xu W, Wu X, Ding X (2025) Life cycle environmental and economic assessment of raw silk production in China. Sustain Prod Consum 55:11–23

[CR67] Xu S, Li Z, Yang Q, Chu G, Zhang J, Zhang D, Zhou H, Gao M (2021) Comparative life cycle assessment of energy consumption, pollutant emission, and cost analysis of coal/oil/biomass to ethylene glycol. ACS Sustain Chem Eng 9:15849–15860

[CR68] Yacout DMM, Abd El-Kawi MA, Hassouna MS (2016) Cradle to gate environmental impact assessment of acrylic fiber manufacturing. Int J Life Cycle Assess 21:326–336

[CR69] Yang P, Chen X, Xu J, Yu L, Ge J, Yang X, Liu J, Zhang S, Zhao F, Feng Y (2025a) Smart flexible fabrics for energy storage, self‐heating, energy harvesting, and self‐powered motion sensing at low temperatures. Adv Funct Mater. 10.1002/adfm.202501330

[CR70] Yang Y, Chen Y, Liu Y, Yin R (2025b) Programmable and scalable embroidery textile resistive pressure sensors for integrated multifunctional smart wearable systems. Adv Fiber Mater. 10.1007/s42765-024-00506-5

[CR71] Zhang Y, Liu X, Xiao R, Yuan Z (2015) Life cycle assessment of cotton T-shirts in China. Int J Life Cycle Assess 20:994–1004

[CR72] Zhang L, Leung M, Boriskina S, Tao X (2023a) Advancing life cycle sustainability of textiles through technological innovations. Nat Sustain 6:243–253

[CR73] Zhang Y, Xia X, Ma K, Xia G, Wu M, Cheung YH, Yu H, Zou B, Zhang X, Farha OK (2023b) Functional textiles with smart properties: their fabrications and sustainable applications. Adv Funct Mater 33:2301607

[CR74] Zhao G, Wen T, Chen C, Wang X (2012) Synthesis of graphene-based nanomaterials and their application in energy-related and environmental-related areas. RSC Adv 2:9286–9303

[CR75] Zhao X, Li J, Dong K, Wu J (2024) Switchable and tunable radiative cooling: mechanisms, applications, and perspectives. ACS Nano 18:18118–18128 38951984 10.1021/acsnano.4c05929

[CR76] Zhao X, Zhang P, Zhang S, Yu W, Wang Z, Hu N, Zhang L (2025) Breathable, robust, and flexible hierarchical design of multifunctional integrated smart textiles for human health management. Chem Eng J 507:160736

[CR77] Zhu S, Liu X (2025) The ecodesign transformation of smart clothing: towards a systemic and coupled social–ecological–technological system perspective. Sustainability 17:2102

[CR78] Zhu L, Zhang W, Luan S, Wei J, Yang Y, Miao J (2025) Nanomaterials for smart wearable fibers and textiles: a critical review. iScience. 10.1016/j.isci.2025.113126 10.1016/j.isci.2025.113126 PMC12341635 40799394

