# Supplementary material for: Life cycle environmental impact of novel radiative thermal management textiles in China
Source: Environ Sci Pollut Res Int. 2026 Mar 16;33(11):5057–68. doi: 10.1007/s11356-026-37632-z (PMC13056727; doi:10.1007/s11356-026-37632-z)
Supplement: Supplementary file 1 — (DOCX 521 KB) [file 11356_2026_37632_MOESM1_ESM.docx]

**Supplementary Information**

**Life Cycle Environmental Impact of Novel Radiative Thermal Management Textiles in China**

Youshan Liang ª, Xiaocheng Hu ^b^, Guanhua Zhang ª, Yu Gong ^c^, Binlin Dou ^a^, Xiaoyu Yan ^b,*^

^a^ School of Energy and Power Engineering, University of Shanghai for Science and Technology, Shanghai 200093, China

^b^ Engineering Department and Environment and Sustainability Institute, University of Exeter, Exeter, UK

^c^ University of Southampton Business School, University of Southampton, SO16 7QB, UK

**^*^** Corresponding authors. E-mail address: [Xiaoyu.Yan@exeter.ac.uk](mailto:Xiaoyu.Yan@exeter.ac.uk) (X. Yan).

**Table of Contents:**

**Text S1. Preparation of functional textiles.**

**Table S1. The electricity consumption of a piece of textile.**

**Table S2.** **The inputs and outputs of 1 kg BN.**

**Table S3. The inputs and outputs of 1 kg MWCNT.**

**Table S4.** **Information of the inventory datasets from Ecoinvent 3.0.**

**Table S5. Explanation of the remaining 12 impact categories.**

**Table S6. The contribution values of a piece of textile from cradle to gate.**

**Table S7.** **Energy consumption for producing different materials for references.**

**Table S8.** **Details of different studied on life cycle assessment of fibers.**

**Table S9. Comparison of the contribution values of the two studies.**

**Figure S1. The picture of prepared textiles.**

**Figure S2. The sensitive analysis of different components.**

**Text S1. Preparation of functional textiles**

The textile is designed based on a dual-sided heterogeneous structure, using polyacrylonitrile (PAN), thermoplastic polyurethane (TPU), and poly (vinylidene fluoride-co-hexafluoropropylene) (PVDF-HFP) as the organic matrix, and combining with inorganic functional particles such as multi-walled carbon nanotubes (MWCNT), silicon carbide (SiC), aluminum oxide (Al₂O₃), and boron nitride (BN). It achieves active switching of the human body surface heating/cooling mode through the coordinated regulation of infrared radiation and solar radiation. Here, taking the fabric synthesis route as an example, and the picture of prepared textiles is shown in Fig. S1.

*Preparation of heating-side spinning solution:* Firstly, 5 g PAN powder were added to a beaker containing 30 mL N,N-dimethylformamide (DMF), and the mixture was stirred at room temperature with a stirrer for 3 hours. The speed of the mixer is set at 400 rpm (the same for the rest of the text). After PAN has completely dissolved in DML, 2 g SiC and 0.5 g MWCNT are added to the mixed solution respectively, and the mixture is stirred for another 2 hours. Finally, the mixed solution was subjected to ultrasonic treatment for 180 s using ultrasonic cleaner to obtain a homogeneous solution without bubbles, which was designated as solution A.

*Preparation of cooling-side spinning solution:* Add 10 g TPU powder and 4 g BN powder to a beaker containing 50 mL DMF, and stir the mixture at room temperature with a stirrer for 5 hours. Finally, the mixed solution was subjected to ultrasonic cleaning for 180 s using an ultrasonic cleaner to obtain a homogeneous and bubble-free mixed solution. Divide the mixed solution evenly into two solutions, B1 and B2. Similarly, prepare solutions C1 and C2 with 6 g PVDF-HFP as the base and doped with 2 g Al_2_O_3_ Nano-powder.

*Textile fabrication:* Using an electrospinning machine to spin thin layers with radiation heat absorption function as the fabric base, and then preparing multi-layer thermal management fabrics by alternately spinning with solutions B1, C1, B2 and C2 on the heating layer base successively. The prepared fabric was first immersed in an ethanol beaker for 12 hours, then soaked in deionized water for another 12 hours, and this process was repeated three times. The soaking process was carried out in a vacuum drying oven at 60℃, and finally the temperature was raised to 80℃ for drying for 24 hours. The injection pump advance speed of solution A is 0.1 mm/min, with a voltage of +20~25 kV, -3 kV. The injection pump advance speed of solutions B and C is 1 mm/min, with a voltage of +20 kV, -3 kV. The needles are all using 17 G.

**Table S1. The electricity consumption of a piece of textile.**

| **Power of electric mixer** | **Total stirring time** | **Electricity consumption of electric mixer** | **Power of electrospinning** | **Total spinning time** | **Electricity consumption of electrospinning** | **Power of drying oven** | **Total using time** | **Electricity consumption of drying oven** | **Power of ultrasonic cleaner** | **Total using time** | **Electricity consumption of ultrasonic cleaner** |
| --- | --- | --- | --- | --- | --- | --- | --- | --- | --- | --- | --- |
| 80 W | 15 h | 1.2 kW·h | 1500 W | 44 h | 66 kW·h | 450 W | 98 h | 44.1 kW·h | 60 W | 0.15 h | 0.009 kW·h |

**Table S2.** **The inputs and outputs of 1 kg BN (**[Lawal Usman et al. 2022](#_ENREF_2)**).**

| **Type** | **Name** | **Amounts** | **Units** |
| --- | --- | --- | --- |
| Inputs | Urea | 1.2 | kg |
|  | Boric acid | 2.5 | kg |
|  | Methanol | 1.4 | kg |
|  | Deionized water | 14.6 | kg |
|  | Average electricity | 500 | kWh |
| Outputs | Waste water | 2 | m^3^ |
|  | Water vapor | 1.8 | kg |
|  | CO_2_ | 0.9 | kg |

**Table S3.** **The inputs and outputs of 1 kg MWCNT (**[Temizel-Sekeryan et al. 2021](#_ENREF_5)**).**

| **Type** | **Name** | **Amount** | **Unit** |
| --- | --- | --- | --- |
| Inputs | Magnesium oxide | 1.1 | kg |
|  | Iron pellet | 0.4 | kg |
|  | Argon | 1.4 | kg |
|  | Acetylene | 0.05 | kg |
|  | Hydrochloric acid | 119 | kg |
|  | Ethanol | 157.9 | kg |
|  | Average electricity | 174 | kWh |

**Table S4. Information of the inventory datasets from Ecoinvent 3.0.**

| **Materials** | **Process** | **Process type** | **Data location** | **Time period** |
| --- | --- | --- | --- | --- |
| PVDF-HPF | Polyvinylfluoride \| market for \| APOS, S | Raw materials | GLO | 2011-2018 |
| Nano-Al_2_O_3_ | Aluminium oxide \| market for \| APOS, S | Raw materials | GLO | 2015-2018 |
| TPU | Polyurethane, flexible foam \| market for polyurethane, flexible foam \| APOS, S | Raw materials | RoW | 2011-2018 |
| PAN | Polyacrylonitrile fibres (PAN), from acrylonitrile and methacrylate, prod. mix, PAN w/o additives EU-27 S | Raw materials | EU | 2005-2012 |
| Nano-SiC | Silicon carbide \| market for \| APOS, S | Raw materials | GLO | 2011-2018 |
| Urea | Urea, as N \| market for \| APOS, S | Raw materials | GLO | 2011-2018 |
| Boric acid | Boric acid, anhydrous, powder \| market for \| APOS, S | Raw materials | GLO | 2011-2018 |
| Methanol | Methanol \| market for \| APOS, S | Raw materials | GLO | 2011-2018 |
| CO_2_ | Carbon dioxide | Raw materials | - | - |
| Magnesium oxide | Magnesium oxide \| market for \| APOS, S | Raw materials | GLO | 2011-2018 |
| Iron pellet | Iron pellet \| market for \| APOS, S | Raw materials | GLO | 2011-2018 |
| Argon | Argon, liquid \| market for argon, liquid \| APOS, S | Raw materials | RoW | 2011-2018 |
| Acetylene | Acetylene \| market for acetylene \| APOS, S | Raw materials | RoW | 2011-2018 |
| Hydrochloric acid | Hydrochloric acid, without water, in 30% solution state \| market for \| APOS, U | Raw materials | RoW | 2011-2018 |
| DMF | N,N-dimethylformamide \| market for \| APOS, S | Raw materials | GLO | 2011-2018 |
| Deionized water | Water, deionised, from tap water, at user \| market for water, deionised, from tap water, at user \| APOS, S | Manufacturing | RoW | 2011-2018 |
| Ethanol | Ethanol, without water, in 95% solution state, from fermentation \| market for \| APOS, S | Manufacturing | GLO | 2011-2018 |
| Electricity | Electricity, low voltage \| market group for \| APOS, S | Manufacturing | CN | 2015-2018 |

**Table S5. Explanation of the remaining 12 impact categories.**

| **Impact category** | **Abbreviation** | **Definition** |
| --- | --- | --- |
| Stratospheric ozone depletion | SOD | Emissions that degrade the stratospheric ozone layer, increasing ultraviolet radiation exposure. |
| Ionising radiation | IR | Potential human exposure to ionising radiation associated with energy and industrial systems. |
| Ozone formation – Human health | OF-HH | Formation of ground-level ozone with adverse effects on human respiratory health. |
| Fine particulate matter formation | FPMF | Generation of fine airborne particles (PM₂.₅) that negatively affect human health. |
| Ozone formation – Terrestrial ecosystems | OF-TE | Ground-level ozone formation causing damage to vegetation and terrestrial ecosystems. |
| Marine eutrophication potential | MEP | Nutrient enrichment of marine systems, primarily driven by nitrogen emissions. |
| Marine ecotoxicity potential | MEP-1 | Potential toxic effects of chemical emissions on marine organisms. |
| Terrestrial ecotoxicity potential | TEP | Toxic impacts of chemical emissions on terrestrial organisms and soils. |
| Human carcinogenic toxicity potential | HCTP | Potential cancer risks to humans from environmental exposure to carcinogenic substances. |
| Human non-carcinogenic toxicity potential | HNCTP | Potential non-cancer human health effects from exposure to toxic substances. |
| Land use | LU | Impacts associated with land occupation and transformation affecting ecosystems. |
| Mineral resource scarcity | MRS | Depletion of mineral resources, reflecting increasing extraction difficulty. |

**Table S6.** **The contribution values of a piece of textile from cradle to gate.**

| **Impact categories** | **Units** | **Total amounts** | **Raw materials acquisition** | **Manufacturing** |
| --- | --- | --- | --- | --- |
| Global warming potential (GWP) | kg CO_2_ eq ^a^ | 125.5 | 3.1 | 122.4 |
| Stratospheric ozone depletion (SOD) | g CFC-11 ^b^ eq | 0.028 | 0.001 | 0.026 |
| Ionizing radiation (IR) | Bq ^c^ Co-60 eq | 2069.6 | 88.5 | 1981.1 |
| Ozone formation-Human health (OF-HH) | g NO_x_ eq | 340.1 | 7.9 | 339.2 |
| Fine particulate matter formation (FPMF) | g PM2.5 eq | 193.8 | 4.9 | 188.9 |
| Ozone formation-Terrestrial ecosystems (OF-TE) | g NO_x_ eq | 347.9 | 8.0 | 339.9 |
| Terrestrial acidification potential (TAP) | g SO_2_ eq | 434.2 | 11.2 | 423.0 |
| Freshwater eutrophication potential (FEP) | g P eq | 24.9 | 0.7 | 24.2 |
| Marine eutrophication potential (MEP) | g N eq | 2.1 | 0.6 | 1.5 |
| Terrestrial ecotoxicity potential (TEP) | kg 1,4-DCB ^d^ | 115.2 | 4.4 | 110.8 |
| Freshwater ecotoxicity potential (FEP-1) | kg 1,4-DCB | 3.99 | 0.09 | 3.9 |
| Marine ecotoxicity potential (MEP-1) | kg 1,4-DCB | 5.0 | 0.1 | 4.9 |
| Human carcinogenic toxicity potential (HCTP) | kg 1,4-DCB | 3.89 | 0.09 | 3.8 |
| Human non-carcinogenic toxicity potential (HNCTP) | kg 1,4-DCB | 44.2 | 1.4 | 42.8 |
| Land use (LU) | m^2^a ^e^ crop eq | 1.6 | 0.1 | 1.5 |
| Mineral resource scarcity (MRS) | g Cu eq | 69.3 | 3.5 | 65.8 |
| Fossil resource scarcity (FRS) | kg oil eq | 24.6 | 0.8 | 23.8 |
| Water consumption (WC) | L | 324.4 | 19.7 | 304.7 |

^a^ eq is the abbreviation of equivalents.

^b^ CFC-11 is the abbreviation of Trichlorofluoromethane.

^c^ Bq is the unit of radioactivity.

^d^ 1,4-DCB is the abbreviation of 1,4 - Dichlorobenzene.

^e^ 1 m^2^a= 1 m^2^*365 day.

**Table S7. Energy consumption for producing different materials for references.**

| **Name** | **Energy consumption (kWh/kg)** | **Required quantity (g)** | **Data source** |
| --- | --- | --- | --- |
| BN | 400-600 | 4 | ([Abdurakhmonov et al. 2025](#_ENREF_1)) |
| MWCNT | 174 | 0.5 | ([Mauron et al. 2003](#_ENREF_3)) |
| Al_2_O_3_ | 3.26 | 2 | Shanxi Huaxing Aluminum Industry Co., Ltd. |
| SiC | 4-5.35 | 2 | ([Riahi et al. 2023](#_ENREF_4)) |
| PAN | 0.28 | 5 | Energy Bureau of Guangdong Province (2020) |
| PVDF-HFP | 2.26 | 6 | Sichuan Zhongfu Taihua New Materials Technology Co., Ltd. |
| TPU | 0.58 | 10 | Energy Bureau of Guangdong Province (2020) |

**Table S8. Details of different studied on life cycle assessment of fibers.**

| **Ref.** | **Products** | **Processes** | **Functional units** |
| --- | --- | --- | --- |
| [Wu et al. (2025)](#_ENREF_6) | Raw silk | Mulberry cultivation - Silkworm farming - Silk reeling | the production of 1 kg of raw silk |
| This work | Composite textile | Raw materials acquisition - Manufacturing | The production of 29.5 g of new prepared textiles |

**Table S9. Comparison of the contribution values of the two studies.**

| **Impact categories** | **Units** | [**Wu et al. (2025)**](#_ENREF_6) | **This work** |
| --- | --- | --- | --- |
| GWP | kg CO_2_ eq | 2.4 | 125.5 |
| SOD | mg CFC-11 eq | 15 | 27.7 |
| IR | kBq Co-60 eq | 0.04 | 2.1 |
| OF-HH | g NO_x_ eq | 3.8 | 347.2 |
| FPMF | g PM2.5 eq | 4.1 | 193.8 |
| OF-TE | g NO_x_ eq | 4.1 | 347.9 |
| TAP | g SO_2_ eq | 17.9 | 434.2 |
| FEP | g P eq | 0.3 | 24.9 |
| MEP | g N eq | 0.04 | 2.1 |
| TEP | kg 1,4-DCB | 6.1 | 115.2 |
| FEP-1 | kg 1,4-DCB | 0.04 | 3.9 |
| MEP-1 | kg 1,4-DCB | 0.06 | 5.0 |
| HCTP | kg 1,4-DCB | 0.05 | 3.9 |
| HNCTP | kg 1,4-DCB | 0.9 | 44.2 |
| LU | m^2^a crop eq | 0.02 | 1.6 |
| MRS | g Cu eq | 6.2 | 69.3 |
| FRS | kg oil eq | 0.6 | 24.6 |
| WC | L | 135.4 | 324.4 |

**Figure S1. The pictures of prepared textile.**


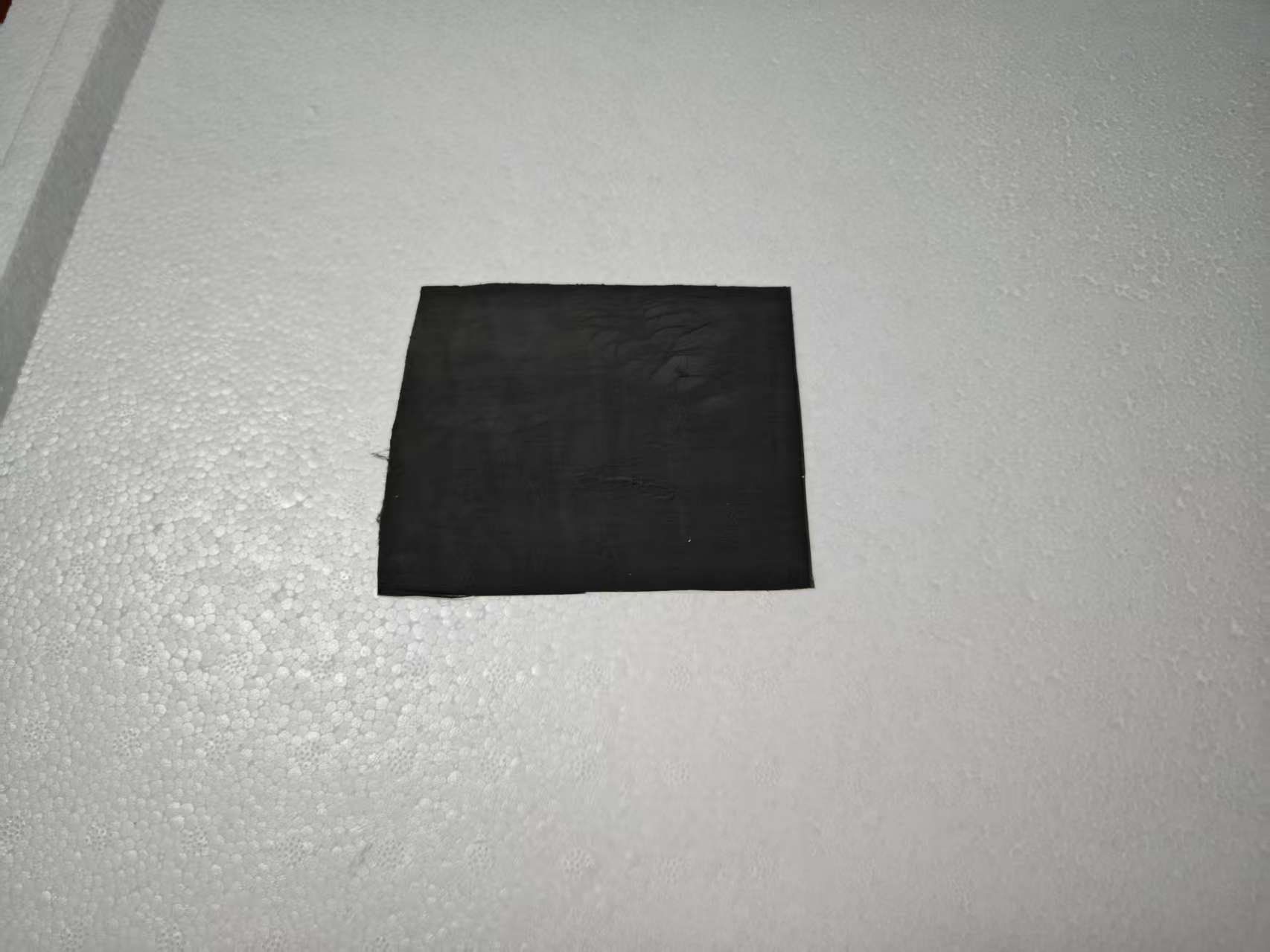

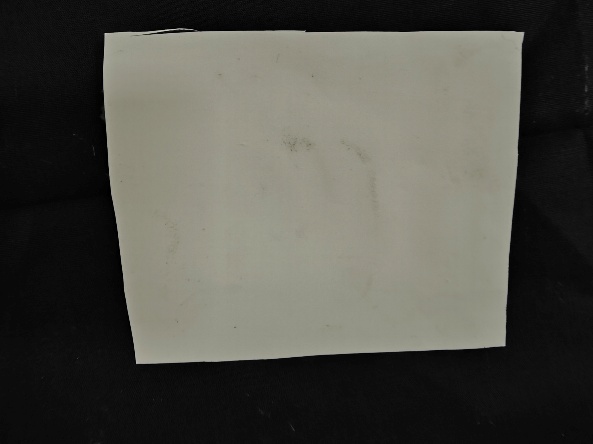


**(a) Heating side**  **(b) Cooling side.**

**Figure S2. The sensitive analysis of different components.**

|  |  |
| --- | --- |
| (a) | (b) |
|  |  |
| (c) | (d) |
|  |  |
| (e) | (f) |
|  |  |
| (g) | (h) |
|  |  |
| (i) | (j) |
|  |  |
| (k) | (l) |

**References**

Abdurakhmonov, O., Aripova, M., Erkinov, F., Abdurakhmonov, S., Sharopov, U., Karimov, M., Kurbanov, M., Saidov, D., Pędzich, Z., Kozien, D., Kurniawan, T. A., Bondar, E., 2025. Green synthesis of high-purity hexagonal boron nitride nanoparticles. Vacuum. 239, 114386.

Lawal Usman, U., Kumar Allam, B., Bahadur Singh, N., Banerjee, S., 2022. Adsorptive removal of Cr(VI) from wastewater by hexagonal boron nitride-magnetite nanocomposites: Kinetics, mechanism and LCA analysis. Journal of Molecular Liquids. 354, 118833.

Mauron, P., Emmenegger, C., Sudan, P., Wenger, P., Rentsch, S., Züttel, A., 2003. Fluidised-bed CVD synthesis of carbon nanotubes on Fe2O3/MgO. Diamond and Related Materials. 12, 780-785.

Riahi, S., McKenzie, J. A., Sandhu, S., Majewski, P., 2023. Towards net zero emissions, recovered silicon from recycling PV waste panels for silicon carbide crystal production. Sustainable Materials and Technologies. 36, e00646.

Temizel-Sekeryan, S., Wu, F., Hicks, A. L., 2021. Global scale life cycle environmental impacts of single- and multi-walled carbon nanotube synthesis processes. The International Journal of Life Cycle Assessment. 26, 656-672.

Wu, Z., Xu, W., Wu, X., Ding, X., 2025. Life cycle environmental and economic assessment of raw silk production in China. Sustainable Production and Consumption. 55, 11-23.
